# The Molecular Apgar Score: A Key to Unlocking Evolutionary Principles

**DOI:** 10.3389/fped.2017.00045

**Published:** 2017-03-20

**Authors:** John S. Torday, Heber C. Nielsen

**Affiliations:** ^1^Pediatrics, Harbor – UCLA Medical Center, Torrance, CA, USA; ^2^Tufts Medical Center, Boston, MA, USA

**Keywords:** Apgar score, water–land transition, adaptation, gene duplication, parathyroid hormone-related protein receptor, β-adrenergic receptor, glucocorticoid receptor

## Abstract

One of the first “tools” used for systematically evaluating successful newborn transitional physiology at birth was the Apgar Score, devised by Virginia Apgar in 1953. This objective assessment tool allowed clinicians to immediately gauge the relative success of a newborn infant making the transition from the *in utero* liquid immersive environment to the *ex utero* gas environment in the delivery room during the first minutes after birth. The scoring system, although eponymous, is generally summarized as an acronym based on Appearance, Pulse, Grimace, Activity, and Respiration, criteria evaluated and scored at 1 and 5 min after birth. This common clinical appraisal is a guide for determining the elements of integrated physiology involved as the infant makes the transition from a “sea water” environment of 3% oxygen to a “land” environment in 21% oxygen. *Appearance* determines the perfusion of the skin with oxygenated blood—turning it pink; *Pulse* is the rate of heart beat, reflecting successful oxygen delivery to organs; *Grimace*, or irritability, is a functional marker for nervous system integration; *Activity* represents locomotor capacity; and, of course, *Respiration* represents pulmonary function as well as the successful neuro-feedback-mediated drive to breathe, supplying oxygen by inspiring atmospheric gas. Respiration, locomotion, and metabolism are fundamental processes adapted for vertebrate evolution from a water-based to an atmosphere-based life and are reflected by the Apgar Score. These physiologic processes last underwent major phylogenetic changes during the water–land transition some 300–400 million years ago, during which specific gene duplications occurred that facilitated terrestrial adaptation, in particular the parathyroid hormone-related protein receptor, the β-adrenergic receptor, and the glucocorticoid receptor. All these genetic traits and the gene regulatory networks they comprise represent the foundational substructure of the Apgar Score. As such, these molecular elements can be examined using a Molecular Apgar evaluation of keystone evolutionary events that predict successful evolutionary adaptation of physiologic functions necessary for neonatal transition and survival.

## Introduction

In the era leading up to newborn intensive care, obstetric anesthesiologists carried the responsibility for evaluating babies in the first few minutes of life and providing resuscitation for those who had difficulty making the transition to extra-uterine life. Dr. Virginia Apgar, an eminent obstetric anesthesiologist at the Columbia University College of Physicians in New York, recognized the need for a method by which doctors and nurses could quickly evaluate and summarize key physiologic factors indicative of the success of the newborn’s transition to extra-uterine existence predictive of subsequent outcomes. She developed a systematic and reproducible assessment approach that predicted the health and well-being of newborns using an easily executed approach. Dr. Apgar published her eponymous “score” in 1953 (1). She selected five physiologic signs that could rapidly be evaluated without difficulty—heart rate, respiratory effort, reflex irritability, muscle tone, and skin color.

At 1 and 5 min after birth a rating of 0, one or two was assigned for each of the five signs, indicating whether it was absent, present but inadequate, or present, yielding a total score between 0 and 10 recorded. She reviewed anesthesia records of 1,025 infants born alive at Columbia Presbyterian Medical Center, New York, NY, USA, at the time of publication of the scoring system. All of the infants in the study had been rated by her method. Infants in “poor” condition had total scores of 0–2, infants in “fair” condition scored 3–7, while total scores of 8–10 were achieved by infants in “good” condition. The most favorable score 1 min after birth was obtained by infants delivered vaginally with the occiput as the presenting part (average Apgar Score = 8.4). Newborns delivered vaginally after *in utero* version or breech extraction had the lowest score (average Apgar Score = 6.3). Infants delivered by cesarean section were more vigorous (average Apgar Score = 8.0) when spinal anesthesia was used versus an average Apgar Score of 5.0 when general anesthesia was used. Correlating the 1-min score with neonatal mortality, Dr. Apgar found that mature infants receiving total scores of 0–2 had a neonatal mortality rate of 14%; those with total scores of 3–7 had a mortality rate of 1.1%; and the mortality in the group with scores of 8–10 was 0.13%. She concluded that the prognosis of the infant is excellent if it attains one of the upper three total scores and poor when the total is one of the lowest three scores.

Before introducing the concept of using the Apgar score to gain insight into evolution it is helpful to consider the perspective of its use by physicians and physiologists. Dr. Apgar’s original purpose was to develop a quick, easily applied, and reproducible summary of a newborn’s transition to extra-uterine life, with the hope that scores would be useful for determining who needed resuscitative measures and would further be a tool for predicting long-term outcomes (1, 2). Numerous studies were performed in subsequent decades by Apgar and many other investigators. Overall, these studies identified the strengths, weaknesses, and limitations of the score. The major strength of the score is that it gives an interpretable snapshot at important post-delivery time points. The snapshot indicates both the state of successful transition and the changes that have occurred since the previous score, i.e., a discriminator of transitional success or lack thereof. However, while the score codifies the momentary status of transition, it is not a useful predictor as Dr. Apgar hoped it would be, even though the 5-min score does correlate with the presence of lactic acidemia and poor neonatal outcome (3). Neither is it a valuable determinator for initiating resuscitation, as resuscitative measures should be in progress well before the 1-min score. Another weakness is that Dr. Apgar had to idiosyncratically assign functions to fit the eponymous nature of the system; this lumps several physiologic events under one heading. As a result, some events, such as “Appearance,” i.e., cyanotic blue versus oxygenated pink, is a code word interpreted as evidence of oxidation in skin capillary beds, and “Pulse,” i.e., heart rate, is a code word for cardiac function interpreted as evidence of appropriate cardiac output and adequate organ perfusion. Each measured score component is susceptible to over-interpretation at the expense of included but less functions not easily or readily measured in the delivery room environment (Schmidt et al.). Thus, when using the Apgar score as a tool for discover of specific evolutionary principles, it is important that the tool be used correctly, applying it with appropriate caution to examine successful evolutionary transitional change.

## From the Apgar Score to Vertebrate Evolution

Virginia Apgar’s revolutionary scoring method has proven durable and successful in part because it finds a symmetry between the adaptive mechanisms the newborn employs to transition from the fetal aquatic-based to the newborn gas-based environment, recapitulating the evolutionary aquatic to land-based adaptation needed for survival. Just as Dr. Apgar’s method summarized the success of the fetal-newborn transition from an aquatic to air-based existence that activated new physiologic events, we can express the success of ancestral life forms making evolutionary transitions that invoke new physiologic functions using a Molecular Apgar Score. Biologically, what Apgar defined was a meaningful, albeit limited, assessment of the manifestations of underlying physiologic adaptations necessary for *ex utero* survival. Recognition of specific biologic relationships between the newborn infant and the ancestral transitioning organisms allows us to identify necessary evolutionary adaptations at the cellular level to address the physiologic challenge that aquatic vertebrates faced in their attempts to transition from water to land some 300–400 million years ago.

Our knowledge of this event is largely based on the fossil record, which reveals that there were at least five attempts to breach land (4). A particular feature of that record is the evidence that the process of land adaptation was characterized by dramatic changes in vertebrate physiology. The transition of ancestral fish from an aquatic environment into terrestrial life forms required major modifications of virtually every organ system to survive in the radically different gaseous environment. Among presumably many genetic modifications, three gene duplication events arose during the Devonian Era, which stand out because of their ability to resolve the existential threat posed by the water–land transition. These are the parathyroid hormone-related protein (PTHrP) receptor (5), the β-adrenergic receptor (βAR) (6), and the glucocorticoid receptor (GR) (7). Reasoning from our post-facto perspective establishes the fact that each of these duplication events was necessary for specific vertebrate adaptations to a land-based ecology.

For the sake of symmetry, we can utilize the same alphabetical mnemonics for the molecular Apgar. In this adaptation of Dr. Apgar’s scoring system, we can identify specific adaptations in molecular genetics that were necessary for the successful transition. It is evident that molecular evolution of PTHrP takes on significant importance. PTHrP is the proxy for “Appearance,” as PTHrP signaling regulates both skin development (8) and vasodilation (9); β-adrenergic effects on the heart, developmentally controlling the cardiac rhythm, and heart rate are represented by “Pulse” (10–12); the developmental and functional integration of the nervous system required for molecular evolutionary changes affecting a wide variety of systems is the “Grimace,” or irritability of Dr. Apgar’s score (13); “Activity” is represented by the development of land-based locomotor function through the exaptation of the role of cholesterol as a precursor to hormone synthesis, energy storage, and ATP production (14); and PTHrP returns to the fore in “Respiration” as a focal center that coordinates the development and function of an adreno-vascular-pulmonary axis necessary for gas exchange in the alveolus. This focal coordination center around PTHrP organizes adrenal output signals from glucocorticoids, cardiovascular β-adrenergic production activation, which are the key elements of land-based respiration (15–17).

We mention here two regulatory pathways subsumed under “Respiration” and “Appearance” that will not be further discussed. The category of “Respiration” includes the central drive for respiration from the medulla and pons. Several neurotransmitters have apparent roles in the feedback control of the respiratory drive. One of particular interest is adenosine and its receptors, the adenosine type 1 and type 2 receptors (AdR-1 and AdR-2), whose activation is responsible for the periodic breathing and apnea that are more prominent in preterm infants but remain features of breathing up to 44 weeks post-conceptional age. AdR-1 and AdR-2 evolved significantly before the water–land transition, during early unicellular adalptation, developing from the primitive G-protein-coupled receptor ancestor, which was likely a cyclic AMP receptor. With development of multicellular organisms AdR-1 and AdR-2 took on increasingly specialized roles in neural network feedback signaling. As neurochemical feedback circuits for respiratory control by AdR-1 and AdR-2 are not evolutionary adaptations specific to the water–land transition, this component of the “Respiration” category is not given further attention.

The nitric oxide pathway is important in controlling vasodilation, important for proper lung perfusion to take up oxygen and for systemic organ perfusion for oxygen delivery. Nitric oxide is produced by nitric oxide synthases (NOS)1, NOS2, and NOS3. Of these, NOS3 is primarily important because, as opposed to NOS1 and NOS2, it is primarily localized to vascular endothelial cells and is induced, providing a rapidly tuneable response. Immediately after birth a significant induction of NOS3 rapidly provides a strong stimulus of pulmonary vasodilation, initiating, and maintaining pulmonary perfusion that enhances oxygen uptake. The phylogenetic evolution of NOS has been extensively defined by several groups (18–20). The evolutionary process is complex, involving at least two episodes each of genomic duplication and gene duplication. While NO and NO-like pathways, primarily mediated by NOS1, have long been present in aqueous non-vertebrates and vertebrates such as fish, NOS3 is present almost exclusively in mammals and only a very few amphibious species. It is apparent that NOS3 did not evolve to facilitate the water–land transition. Likely, it was co-opted much later than this evolutionary event to further facilitate survival after live birth.

## Evolutionary Adaptations in the Land–Water Transition: The Apgar Perspective PTHrP and Integrated Physiology

The transition from an aquatic-based to a land-and gas-based environment exposed the animal to several new challenges to survival. These included (1) increased gravitational effects on the body, requiring a more robust skeletal system; (2) exposure to a greater variety of sources of molecular damage through exposed surfaces, requiring specific epithelial changes to protect the skin, gut, and lungs from external antigens and energy forms; and (3) increased energy needs, especially to increase metabolic efficiency, necessitating ectotherms to develop into endotherms. These needs were met by functions assumed by PTHrP and its receptor. Integrated vertebrate physiology is prominent largely because of its ancient utility in adapting to the force of gravity, the oldest, most ubiquitous, and constant force exerted on life on earth (21, 22). PTHrP also addressed the adaptive needs for protection against a harsher external environment and for increased energy supply, in the latter instance by increasing the supply of oxygen, the necessary catalyst for energy production. In this context, it is noteworthy that NOS3 activation is a down-stream effect of PTHrP signaling, consistent with an evolutionary adaptation subsequent to the land–water transition to further improve post-birth survival. PTHrP and its receptor are mechanotransducers that are regulated by physical distention (23). Deletion of PTHrP in mice results in major disruptions of the evolutionary pressure-driven adaptations: the lung does not form alveoli (15); bone ossification fails to occur (24); and skin does not develop (25). Each of these traits was essential for successful vertebrate adaptation to land. In addition, deletion of PTHrP affects the kidney (26) and brain (27). PTHrP is expressed throughout the body in all epithelial cells (28); its receptor is located on neighboring mesodermal cells. This juxtaposition promotes local influences on morphogenesis (29), culminating in homeostasis. Disruption of these homeostatic mechanisms results in a range of disorders relative to successful land adaptation, from defective development leading to early neonatal mortality, to chronic disease and fibrotic scarring (30, 31). In a variety of organs, some pathways influenced by PTHrP are regulated by stretch, including the lung (23), kidney (32), bone (33), and uterus (34), reflecting the need to accommodate gravitational forces (35). Under microgravitational conditions, lung and bone homeostasis are disrupted (36), causing structural and functional damage. This likely reflects their ancestral roles in adaptation to an atmospheric land-based environment, namely air breathing and skeletal support. Many other PTHrP-expressing tissues and organs are not as structurally and functionally impacted by deletion of PTHrP. It is possible that these are more recent derivatives of the ancestral traits that co-evolved as a result of the efficacious PTHrP receptor gene duplication associated with a successful evolutionary water–land transition.

## βAR Gene Duplication and Vascular Adaptation

Evolution of the lung, including the development of the pulmonary microvascular circulation independent of the systemic circulation, was a response to the developmental pressure of increased oxygen demands for thermoregulation. This allowed expansion of both the gas-exchange epithelial surface area and the investing capillary bed (37). Moreover, it required a significantly increased cardiac output resulting from the progressive development from a linear into a parallel cardiovascular circuitry. The parallel circuitry of the four chambered heart created further evolutionary pressure on control of metabolic demands. It is likely that duplication of the βAR increased the capability of the organism to successfully transition from a poikilothermic water environment to a homeothermic atmospheric environment. This adaptation is a significant component represented by the “P” in the evolutionary Apgar score.

Without the increase in βARs in the alveolar microvasculature, alveolar evolution alone would not be sufficient to support the metabolic demands of land vertebrates (17), since the capillaries of the alveoli would have been repetitively damaged by progressive increases in systemic blood pressure due to physiologic stress conditions. The increased population of the blood vessels by βARs may also have facilitated the linear to parallel evolution of the heart. This evolutionary progression is phylogenetically evident, going from one chamber in worms, to two chambers in fish, to three chambers in frogs, and four chambers in quadrapeds in tandem with lung evolution (38). Deletion of the βAR gene in mice interferes with cardiac morphogenesis in a pattern suggestive of evolutionary regression, creating only the two-chambered heart characteristic of fish (10). The complementary evolution of the lung and heart, promoted by the tandem evolution of the PTHrP receptor and the βAR, strongly suggests a co-evolutionary adaptation in response to the same pressures (38), particularly when considering the integrated, functional “cross-talk” between the heart and lung fostering positive selection for such mechanisms.

## GR Gene Duplication

The GR evolved from the mineralocorticoid receptor (MR). A driving force underlying this adaptation was likely the constraint of the increased effect of gravity with the transition from water to land (39) driving the need for a mechanism to regulate orthostasis-related changes in blood pressure. Gravity’s environmental pressure was compounded by mineralocorticoid stimulation of blood pressure (40), now offset by diverting some of the MR expression to the GR by adding two amino acids to the MR (7). This adaptation, combined with the synergistic effect of adrenocortical glucocorticoid production on adrenomedullary βAR production (41, 42) also relieved hypoxic stress at the level of the alveoli by distending the alveolar walls, thereby increasing the capacity for oxygen uptake to meet metabolic demands. The “Pulse” component of the evolutionary Apgar score therefore represents the adaptive changes that allow adrenaline to regulate cardiac supply by controlling both heart rate and vascular tone.

## Evolution of Endothermy/Homeothermy: Evidence of the Effect of Environmental Stress on Vertebrate Physiologic Evolution

One can readily propose the counter hypothesis that none of these physiologic changes reflect an evolutionary causation since there is no physical fossil evidence for this sequence of events. However, this ignores the reality that these functional interrelationships are undeniably consistent with their contemporary roles in ontogenetically generating and phylogenetically sustaining homeostasis. A causal relationship of these mechanistic adaptations with phylogeny is arrived at in a number of ways by which it is recognizable that the changes of these physiologic traits are internally consistent, particularly when considered from the perspective of the components of the evolutionary Apgar score. Initial evidence is derived by considering the phenotype resulting from deletion of either the βAR (10–12) or the GR (43). Furthermore, an organismal consideration of the mutually interdependent nature of these evolutionary changes reveals that changes of these physiologic traits are internally consistent with their ontogeny and phylogeny through the advent of endothermy/homeothermy (44), a position that has been frequently emphasized in this essay.

**Figure 1:**
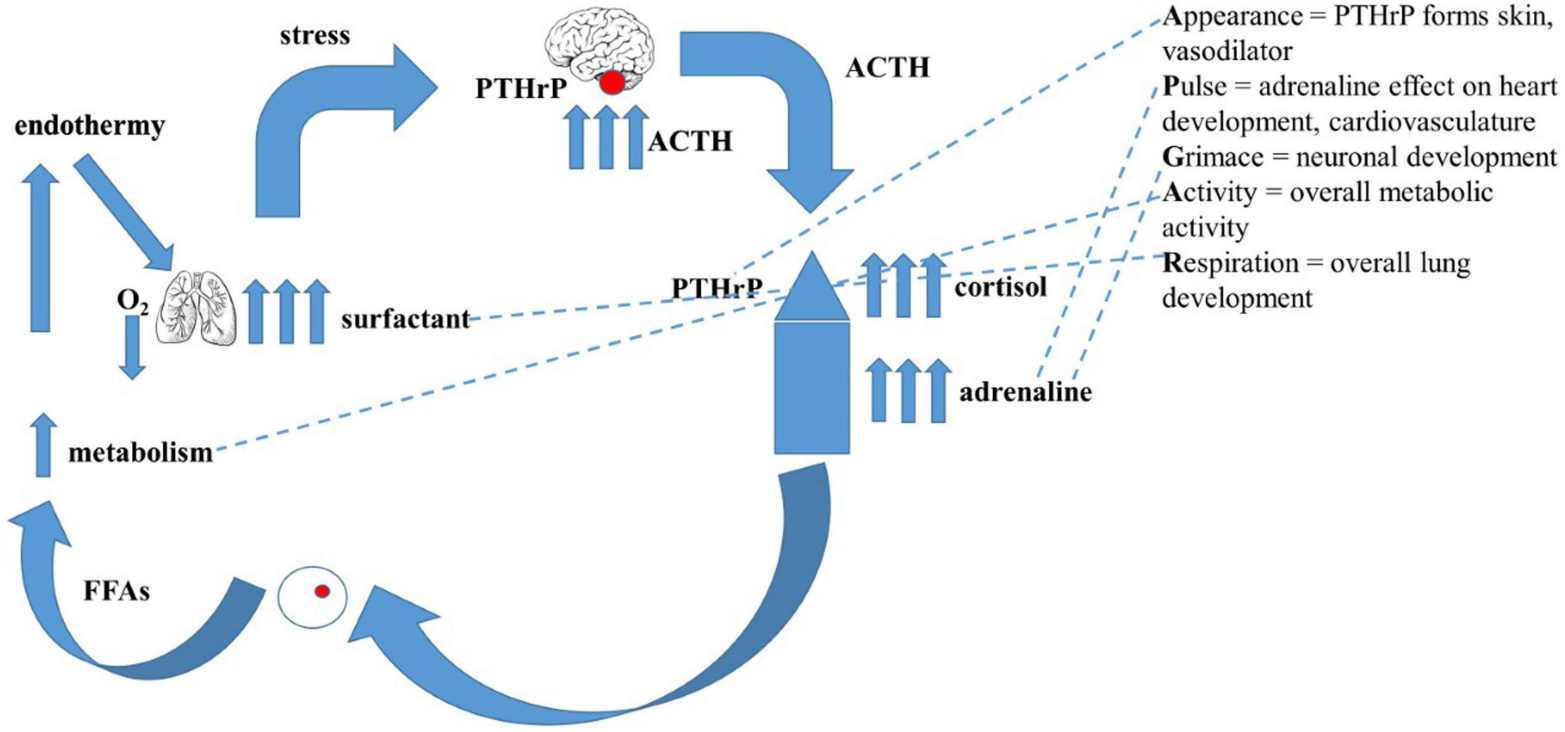
**The evolutionary basis for the “Molecular Apgar Score.”** On the left is depicted the evolution of endothermy: intermittent hypoxia due to pulmonary’ insufficiency during lung evolution would have stimulated catecholamine production by the adrenals, acutely resolving the constraint on air breathing by stimulating alveolar surfactant production, rendering the alveoli more distensible, increasing oxygenation. A collateral effect of increased catecholamines on the secretion of fatty acids from peripheral fat cells would consequently have increased both basal metabolic activity and body temperature. On the right are the homologs of these steps in the evolution of endothermy seen as the properties of the Apear Score. Appearance = PTHrP forms skin, vasodilator; Pulse = adrenaline effect on heart development, cardiovasculature; Grimace = neuronal development; Activity = overall metabolic activity; Respiration = overall lung development.

A non-teleological mechanism for the evolution of endothermy/homeothermy has not previously been proposed (44). By exploiting the abovementioned gene duplications, a mechanism of developmental adaptations of pre-existing physiologic traits in response to evolutionary pressures that gave rise to endothermy/homeothermy has been developed (44). Briefly, intermittent hypoxia due to pulmonary insufficiency during lung evolution stimulated catecholamine production by the adrenals (45). This acutely resolved the limiting constraint on the effectiveness of air breathing by stimulating alveolar surfactant production (46), rendering the alveoli more distensible and thereby increasing oxygenation (47). A parallel resolution of the constraint imposed by ambient temperature fluctuations was attained by increased catecholamine production and signaling to enhance the secretion of fatty acids from peripheral fat cells (48), consequently increasing both basal metabolic activity (49) and body temperature (50). Figure 1 depicts how these evolved physiologic traits for adaption to land are represented in the molecular Apgar Score.

Leptin contributed a further endothermic/homeothermic solution to the constraining pressure of body temperature regulation. The evidence that leptin increases the basal metabolic rate of cold-blooded Fence Lizards (51), for example, gives leptin a role supportive of catecholamines in the evolutionary response that resolved ambient temperature constraints through development of effective endothermy. The increased and self-regulated body temperature also synergized with the thermal chemistry requirements of evolved mammalian lung surfactant. The major component of mammalian lung surfactant is the species of phosphatidylcholine, which contains a saturated fatty acid at the sn-1 and sn-2 positions of its triglyceride backbone (saturated phosphatidylcholine). Saturated phosphatidylcholine functions three times more efficiently to decrease surface tension at 37°C than at ambient atmospheric temperature (25°C) (52). This property is due to the elevated phase transition temperature of saturated phosphatidylcholine (41°C), the temperature at which the surface film formed by lung surfactant to decrease surface tension collapses such that it no longer reduces surface tension (53).

The co-evolution of saturated phosphatidylcholine production by the alveolar epithelium and endothermy/homeothermy may have been promoted by catecholamine’s pleiotropic activity that both stimulates surfactant secretion in the alveoli and complementarily increases the fractional proportion of unsaturated phospholipids populating peripheral cell membranes (54). The preponderance of unsaturated phospholipids in cell membranes promotes cellular oxygen uptake by increasing membrane fluidity (55). Thus, the phylogenetic increased proportion of saturated phosphatidylcholine in lung surfactant (56, 57) required a successful adaptation to endothermic/homeothermic body temperature regulation. Such fundamental changes in lipid composition in furtherance of metabolism are exaptations of the events that initiated eukaryotic evolution (16). In hindsight, given the global constraints caused by Romer’s Gap (7), during which most of the land vertebrate species were eradicated, it is not surprising that such deep homologies would evolve to sustain survival during this critical phase of vertebrate evolution.

## Hibernation as the Mirror Image of Physiologic Stress

The coordinate effects of hibernation on lung surfactant lipid composition and cell membrane fatty acid composition illustrate the causal interrelationship between hypoxia, physiologic stress, catecholamines, and endothermy/homeothermy. The decreased physiologic stress of hibernation results in lowered adrenomedullary catecholamine production, causing increased lung surfactant cholesterol content (58, 59), rendering the surfactant less surface active. The lowering of catecholamine production also causes decreased unsaturated fatty acid content of peripheral tissue cell membranes (60), adaptively reducing oxygen uptake.

There is experimental evidence for the effect of environmental temperature changes on lung surfactant phospholipid fatty acid composition. Lau and Keough (61) maintained Map turtles at different ambient temperatures, altering the composition of their lung surfactant in adaptation to the prevailing environmental conditions. Such adaptations to environmental temperature change may ultimately have given rise to endothermy, given the evidence for the causal interrelationships between body temperature (61), surfactant composition (61), and catecholamine regulation of surfactant secretion (46).

The enabling effects of cholesterol on cell membrane function from the inception of unicellular eukaryotic evolution provide the basis for the role of lipids in accommodating endothermy (16). The evolution of the alveolar lipofibroblast in mammals attests to the fact that these are not merely associations. Lipofibroblasts are adipocyte homologs that are located within the alveolar wall next to the alveolar epithelial cells that produce surfactant (62), providing substrate locally for “on demand” surfactant phospholipid production in compliance with the physiologic need for oxygen by way of the stretch-regulated mechanism described above. In further support of the role of lipofibroblasts in alveolar lung evolution, when cholesterol synthesis by alveolar type II cells was genetically deleted from the embryonic mouse lung alveolar type II cell (63), lipofibroblast proliferation compensated for the loss of surface tension reducing capacity (63). This compensatory effect was due to increased peroxisome proliferator-activated receptor gamma expression by these cells (64) due to endoplasmic reticulum stress (64), recapitulating the mechanism by which peroxisomes originally evolved (65). Such ancestral traits were exploited to generate the molecular Apgar Score.

Similarly, an integrated cascade of physiologic stress-mediated cellular mechanisms gave rise to the kidney glomerulus. Phylogenetically, fish do not have glomeruli, but amphibians, reptiles, mammals, and birds all do (66). PTHrP mediates fluid and electrolyte balance in the glomerulus. It is secreted by the podocytes that line the glomerulus, binding to PTHrP receptors on the mesangium, regulating the fluid and electrolytes entering the kidney tubules (67); like the lung alveolar type II cells, the podocytes sense the distension of the glomerulus, transducing that stretching signal for fluid and electrolyte balance *via* PTHrP signaling. This homology between such structurally and functionally disparate tissues and organs as the lung and kidney exemplifies the pleiotropic distribution of the same cellular–molecular mechanism in service to both gas exchange and fluid and electrolyte balance. These convergent evolutionary traits may also have evolved under positive selection for increased catecholamine production under physiologic stress because epinephrine inhibits the loss of water and salt from the kidney (68) in adaptation to land.

## Predictive Power of the Cellular–Molecular Approach to the Apgar Score

Starting with the unicellular perspective of the life cycle as the primary level of selection (44), and the need to iteratively return to it as a necessity for identifying and understanding adaptive strategies for epigenetic inheritance, the cellular–molecular approach is highly predictive in comparison to the conventional descriptive approach to biology that has been the rule for hundreds of years. The recognition that the cell membrane is the homolog for all complex physiologic traits (44) forms the basis for understanding the first principles of physiology (69). And by focusing on the mechanistic transition from the unicellular state to the multicellular organism during both ontogeny and phylogeny, such seemingly enigmatic properties of life as pleiotropy (70), the stages of the life cycle (70), and the aging process (16) can all be understood as one continuous process of emergence and contingence.

## Conclusion

Devising an Apgar Score based on evolutionary principles would provide the basis for a predictive model of physiology. By focusing on lipids in initiating and facilitating the evolution of eukaryotes (16), a vertically integrated perspective for ontogeny and phylogeny becomes tenable. The advent of cholesterol in the cell membrane of unicellular eukaryotes formed the basis for vertebrate evolution by fostering metabolism, gas exchange, locomotion, and endocytosis/exocytosis (16). Prokaryotes and eukaryotes differ in the hard exterior of the former versus the soft, compliant cell membrane of the latter. As a result, eukaryotes are better suited for more complex interactions with the external environment. They adapt by internalizing factors in the environment and compartmentalizing them to form physiologic systems, from the cell to the organism. Competition with prokaryotes set this process in motion, since bacteria can emulate such pseudo-multicellular behaviors as Biofilm (71) and Quorum Sensing (72). Such physiologic traits as those gained during the water–land transition, lipofibroblasts, endothermy/homeothermy, and peroxisomes are fractals of the originating principle of lipids in service to the evolution of eukaryotes. Careful scrutiny of the evolutionary Apgar Score reveals the core roles of lipids in biologic mechanisms.

Seen from its unicellular origins instead of its overt present day phenotypic appearances and functional associations provides a robust, predictive picture of how and why complex multicellular vertebrate physiology evolved from unicellular organisms. This approach lends itself to a deeper causal understanding of what the Apgar Score is actually measuring at the genomic level when applied to newborn infants. The Apgar Score is a surrogate for the cellular–molecular changes that facilitated vertebrate adaptation to land. The reasons for such processes as the life cycle and why organisms return to their unicellular state emerge from such a cellular–molecular perspective.

There has been tension between calcium and lipid homeostasis ever since the inception of life (16). Such tension has been alleviated by the formation of calcium channels from those self-same lipids (73), providing a common evolutionary strategy. The consequent rise in atmospheric carbon dioxide (74) generated carbonic acid when dissolved in water, causing increased calcium content of water by leeching it from the rock. Calcium ion fluxes are necessary for all vertebrate metabolism (16); the inception of life is marked by a calcium burst triggered by sperm fertilizing the ovum (75). That flow of calcium sustains the processes of life until the moment of death (76). The aura of light seen during near-death experiences (77) may be that last calcium burst of the life force before death.

A mechanistically cohesive, vertically integrated view of physiology has long been sought. Lancelot Whyte described it as Unitary Biology (78), but his concept had no scientifically causal basis, so it remained philosophical speculation. However, with the discovery of soluble growth factor signaling as the mechanistic basis for embryonic pattern formation in 1978 (79), Whyte’s hope of a “singularity” became feasible (78). Thus, the fundamental difference between descriptive and mechanistic physiology, particularly with reference to homeostatically regulated epistatic balance between calcium and lipids, has been highlighted. Moreover, the mechanisms underlying the Apgar Score emblematic of the self-organizing (80), self-referential (81) nature described for the origin of life itself are highly relevant. Centering on such organizing principles avoids the perennial pitfalls that teleology leads to (82). Instead, such oppositional dichotomies as genotype–phenotype, emergence–contingence, and unicellular–multicellular organisms are resolved. Resolution of the fundamental interrelationship between calcium and lipid homeostasis by cellular communication was first chronicled in “Evolutionary Biology, Cell–Cell Communication and Complex Disease” (16). The utility of focusing on the advent and roles of cholesterol in eukaryotic evolution will be shored up by further investigation of the gap between unicellular and multicellular organisms. Such research will provide novel insights to the true nature of the evolutionary continuum in a predictive manner. Such fundamental understanding of the “how and why” of evolution provides the unprecedented basis for developing a Central Theory of Biology (44). Many have previously given up on a predictive model for biology like those for chemistry or physics. The failure to recognize that biology is descriptive is at the at the core of this failure, misconstruing describing a mechanism being the same as actually determining causation based on founding principles, as has been achieved through quantum mechanics and general relativity theory. In light of the publication of the Human Genome, it seems surprising that biology remains descriptive. However, the unexpected finding that the human genome is smaller than a carrot should have generated a critical re-evaluation of the prevailing approach to biology as a *fait accompli*, characterized by correlations and associations. John Ioannidis has declared that “most published research findings are false” (83). This may be because the prevailing biologic approach is based on a descriptive framework that generates associations and correlations, not predictions. The molecular Apgar Score, as presented here, has successfully generated predictions of the mechanisms by which the water–land transition was evolutionarily accomplished.

## Ethics Statement

Authors are required to state the ethical considerations of their study in the manuscript including for cases where the study was exempt from ethical approval procedures. Did the study presented in the manuscript involve human or animal subjects: no.

## Author Contributions

JT and HN contributed equally to this manuscript.

## Conflict of Interest Statement

The authors declare that the research was conducted in the absence of any commercial or financial relationships that could be construed as a potential conflict of interest.
